# Detection of Salivary Small Extracellular Vesicles Associated Inflammatory Cytokines Gene Methylation in Gingivitis

**DOI:** 10.3390/ijms21155273

**Published:** 2020-07-24

**Authors:** Pingping Han, Andrew Lai, Carlos Salomon, Sašo Ivanovski

**Affiliations:** 1 School of Dentistry, The University of Queensland, Herston, QLD 4006, Australia; 2 Exosome Biology Laboratory, Centre for Clinical Diagnostics, The University of Queensland Centre for Clinical Research, Royal Brisbane and Women’s Hospital, The University of Queensland, Brisbane, QLD 4029, Australia; a.lai@uq.edu.au (A.L.); c.salomongallo@uq.edu.au (C.S.); 3 Department of Obstetrics and Gynecology, Ochsner Baptist Hospital, New Orleans, LA 70422, USA; 4 Department of Clinical Biochemistry and Immunology, Faculty of Pharmacy, University of Concepción, 4030000 Concepción, Chile

**Keywords:** salivary small extracellular vesicles, ultracentrifuge, size exclusion chromatography, DNA methylation, cellular uptake

## Abstract

Salivary small extracellular vesicles (sEV) are emerging as a potential liquid biopsy for oral diseases. However, technical difficulties for salivary sEV isolation remain a challenge. Twelve participants (five periodontally healthy, seven gingivitis patients) were recruited and salivary sEV were isolated by ultracentrifuge (UC-sEV) and size exclusion chromatography (SEC-sEV). The effect of UC and SEC on sEV yield, DNA methylation of five cytokine gene promoters (interleukin (IL)−6, tumor necrosis factor (TNF)-α, IL−1β, IL−8, and IL−10), and functional uptake by human primary gingival fibroblasts (hGFs) was investigated. The results demonstrated that SEC-sEV had a higher yield of particles and particle/protein ratios compared to UC-sEV, with a minimal effect on the detection of DNA methylation of five cytokine genes and functional uptake in hGFs (*n* = 3). Comparing salivary sEV characteristics between gingivitis and healthy patients, gingivitis-UC-sEV were increased compared to the healthy group; while no differences were found in sEV size, oral bacterial gDNA, and DNA methylation for five cytokine gene promoters, for both UC-sEV and SEC-sEV. Overall, the data indicate that SEC results in a higher yield of salivary sEV, with no significant differences in sEV DNA epigenetics, compared to UC.

## 1. Introduction

Small extracellular vesicles (sEV) are nano-scaled (<200 nm), stable lipid bilayer membrane surrounded structures that are abundant in biofluids (e.g., saliva and plasma) [1]. sEV have attracted considerable interest as a liquid biopsy source for the discovery of biomarkers, as their cargos (RNA, DNA, and protein) can be considered to be biomolecular “fingerprints” of the releasing cell and its metabolic status [1,2,3,4,5]. Recent reports indicate that salivary sEV are potential diagnostic tools for local conditions such as oral cancer [6], as well as systemic diseases such as inflammatory bowel disease [7] and lung cancer [8]. However, the roles of salivary sEV in oral and periodontal health are not well understood, and there is virtually no information on sEV inflammatory genes DNA methylation in oral health and disease.

Salivary sEV isolation methodology remains challenging and is still developing, due to the interference of highly abundant viscous proteins present in saliva [9]. Currently, two main methods have been reported to isolate salivary sEV, namely the ‘gold-standard’ ultracentrifuge (UC) and a commercial ExoQuick (EQ) precipitation kit [10]. Alternatively, size exclusion chromatography (SEC) has been proposed for sEV isolation with potential higher functionality [11] and was recently used to isolate salivary sEV [12]. Thus, evaluating the different techniques (UC vs. SEC) is of great importance for establishing an optimal method for salivary sEV isolation and understanding salivary sEV characteristics.

Gingivitis is a highly prevalent inflammatory oral condition associated with a bacterial dental polymicrobial biofilm [13]. The biofilm may predispose patients to the more severe and destructive periodontal disease periodontitis. Hence, for periodontal diagnosis and prognosis, it is of importance to detect periodontal pathogen-byproducts (genomic DNA-gDNA) among salivary sEV populations. Furthermore, since periodontal diseases are inflammatory medical conditions in nature, it is not surprising that increased salivary inflammatory cytokines, such as IL−6, TNF-α, IL−1β, IL−8, and IL−10, are associated with gingivitis [14]. Notably, oral health status is rarely considered in salivary diagnostics research and more work is required to understand the impact of periodontal disease status on salivary biomarkers, especially those associated with inflammation.

It is noted that inflammatory disease may be associated with epigenetic regulation, in particular DNA methylation [15], which is characterized by an additional methyl modification at 5′ cytosine within the CpG regions. DNA methylation in the promoter regions of specific genes can activate or inactivate gene expression [16]. Recent research demonstrated that cytokine gene expression is associated with DNA methylation changes within saliva [17]. However, DNA epigenetic changes in gingival tissues or blood have generally been assessed in invasive tissue biopsy samples [15,18], with fewer attempts at utilizing saliva-derived sEV as an alternative sampling source.

Human gingival fibroblasts (hGFs) are the main cells within gingival connective tissue [19]. It is therefore of interest to determine the effect of salivary sEV on the function of hGFs, with an emphasis on cellular DNA methylation. Recent research findings suggest that CD9 positive salivary sEV are decreased in periodontitis patients when compared to periodontally healthy controls [10]; with a limitation of this study being the lack of a gingivitis group and the sEV being isolated using the EQ method. Another study compared saliva sEV isolation by UC and EQ and it showed that CD9 and CD81 expression was increased in EQ-sEV compared to UC-sEV [20]. Our very recent research demonstrated that SEC-sEV associated miRNAs (hsa-miR−140–5p, hsa-miR−146a−5p, and hsa-miR−628–5p) were only detected in sEV in periodontitis when compared to that of healthy controls [12].

**Figure 1:**
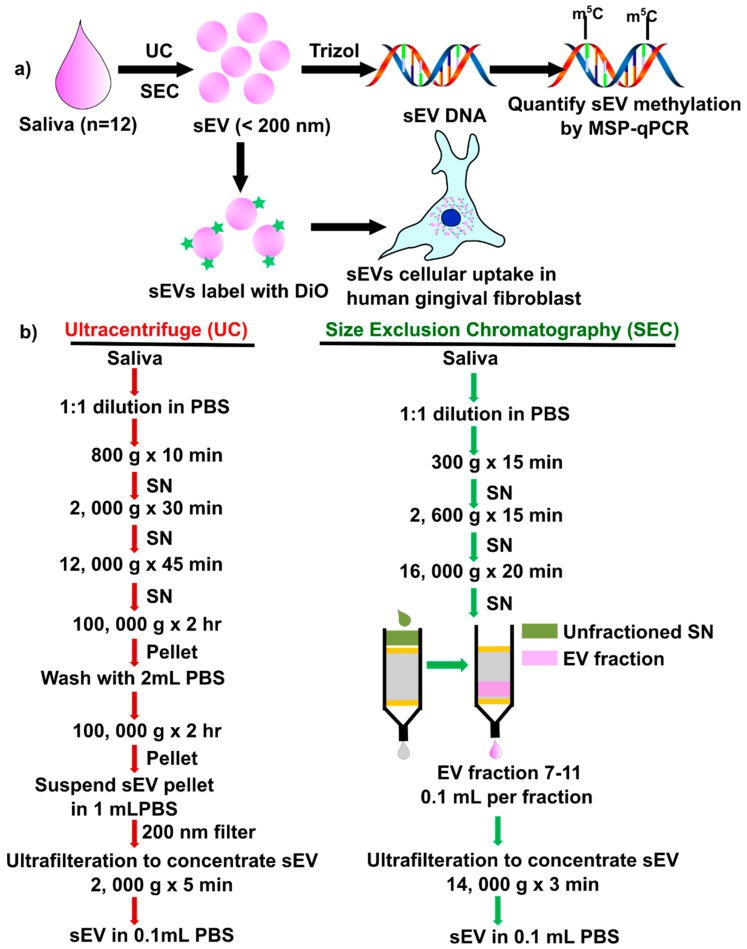
Schematic diagram of the study design (**a**) and detailed methods (**b**) to isolate salivary small extracellular vesicles (sEV) by ultracentrifuge (UC) and size exclusion chromatography (SEC). DiO: 3,3′-Dioctadecyloxacarbocyanine perchlorate; qMSP: quantitative methylation-specific real-time PCR; SN: supernatant.

There are no reports to compare sEV isolations method (UC vs. SEC) on the yield, periodontal pathogens, DNA methylation of inflammatory cytokine genes, and functional activity of salivary sEV in hGFs. In this study, we compared two salivary sEV isolation methods, using ultracentrifuge (UC method: UC-sEV) and an SEC column (SEC method: SEC-sSEV) and analyzed their impact on inflammatory cytokine gene methylation and hGFs uptake (Figure 1a). The results demonstrated that SEC is a reliable isolation method, with higher sEV particles yield, while there was no significant difference between UC and SEC-sEV DNA methylation patterns.

## 2. Results

### 2.1. Periodontal Status of Participants

As shown in Table 1, the participants (*n* = 12) were from various ethnic backgrounds (50% Caucasian, 42% Asians, and 8% other) and mixed gender (eight males, four females), with an age range from 23 to 48. No participant had periodontitis, defined as presenting with periodontal pocketing of 4 mm or higher. Bleeding on probing (BOP) ranged from 6% to 70%, with five participants being less than 15% (defined as healthy according to the latest periodontal disease classification [21]), while seven were over 15% (defined as gingivitis).

### 2.2. Particle and Characteristics of Salivary sEV by UC or SEC

Salivary sEV were isolated using the UC method and SEC method from all 12 participants, according to the method described in Figure 1b. A commercial lyophilized salivary exosome (sEV) standard from ‘healthy’ donors (cat#: HBM-PESL; HansaBioMed, Lonza) was used as a control. HansaBioMed’s purified lyophilized exosomes are isolated through a combination of ultracentrifugation and microfiltration procedures.

**Figure 2:**
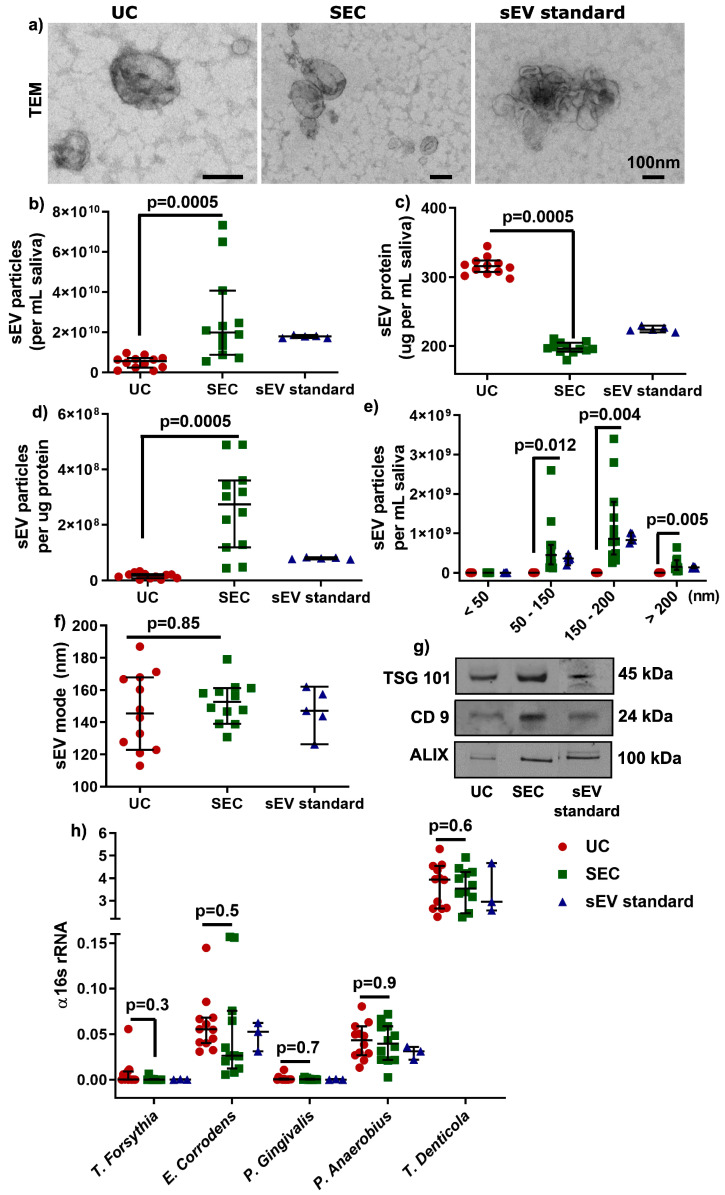
Characterization of salivary sEV by transmission electron microscopy (TEM) (**a**), NTA (**b**–**f**), and Western blot (**g**). Detection of periodontal pathogens gDNA in UC- and SEC-sEV (**h**). Western blot bands in (**g**) were cropped from different lanes of the same gel (Supplementary Figure S2).

According to the latest international EV research guidelines [22], sEV were characterized by morphology with transmission electron microscopy (TEM, Figure 2a), by particle concentration and size distribution (<200 nm, Figure 2b–f) using Nanoparticle Tracking Analysis (NTA), and by sEV-enriched protein markers (Tumor susceptibility gene 101 protein (TSG 101), CD9, and ALG-2 interacting protein X (ALIX), Figure 2g) with Western blot. sEV particles per mL saliva were significantly higher (≈5 times more) in SEC-sEV compared to UC-sEV (2.7 × 10^10^ vs. 5.07 × 10^9^; Table 2 and Figure 2b), with less protein concentration (Figure 2c), higher particles/protein ratio (Figure 2d), and significantly higher particles for the size ranges of 50–150 and 150–200 nm (Table 2 and Figure 2e). Furthermore, the mode of sEV sizes were less than 200 nm and comparable between the UC and SEC isolation method (Figure 2f).

Moreover, sEV-associated gDNA and RNA (from ≈10^9^ particles) were comparable in quantity and quality between UC and SEC (Supplementary Figure S1), although the overall quality of DNA and RNA could be improved. Additionally, the gDNA-qPCR results showed that there was no significant difference found between UC and SEC-sEV for the periodontal Gram-negative bacteria strains: *Tannerella forsythia (T. forsythia), Eikenella corrodens (E. corrodens), Porphyromonas gingivalis (P. gingivalis), Peptostreptococcus anaerobius (P. anaerobius),* and *Treponema denticola (T. denticola*) (Figure 2h), suggesting periodontal pathogens could be detected for both UC- and SEC-sEV.

Overall, the results showed that the SEC method could lead to more enriched sEV particles compared to the UC method. There was no significant difference in periodontal bacterial gDNA detection between UC and SEC.

### 2.3. Effect of UC and SEC Isolation Methods on Healthy and Gingivitis-Derived sEV Characteristics

**Figure 3:**
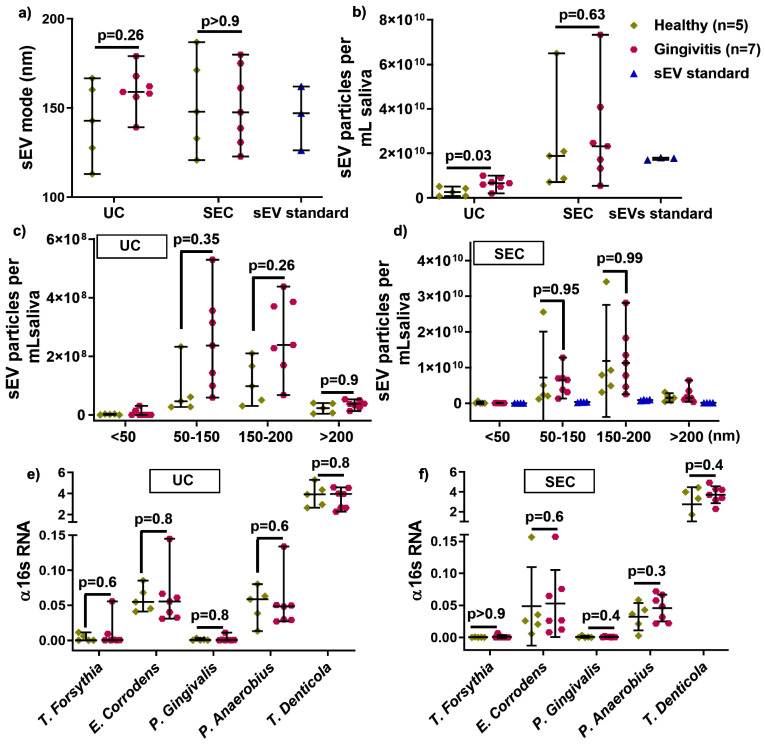
The influence of UC and SEC method on healthy and gingivitis sEV characteristics, such as size (**a**), particle concentration in per mL saliva (**b**), size distribution (**c**,**d**), and periodontal pathogen OMVs (**e**,**f**).

To assess the effect of UC and SEC on healthy (*n* = 5) and gingivitis (*n* = 7) derived sEV, the average size, particle concentration, size distribution, and bacterial strains were analyzed. There was no size difference between healthy and gingivitis-derived sEV in either UC and SEC isolated samples (Figure 3a). The UC method led to significantly increased particle numbers in gingivitis participants (*p* = 0.03) compared to that of healthy controls, while the numbers were comparable between health and gingivitis for the SEC method, albeit substantially higher than UC for both disease states (Figure 3b). Furthermore, no significant difference was detected between the healthy and gingivitis groups for all of the size ranges (< 50, 50–150, and 150–200 nm) for both UC and SEC (Figure 3c,d). 

To investigate the effect of UC and SEC on human and bacteria-sourced sEV during salivary sEV isolation, gDNA-qPCR was used to compare healthy and gingivitis-associated sEV. Periodontal diseases-associated bacterial strain (*T. forsythia*, *E. corrodens*, *P. gingivalis*, *P. anaerobius*, and *T. denticola*) were comparable in healthy and gingivitis-sEV from both the UC and SEC isolation methods (Figure 3e,f).

### 2.4. UC and SEC on sEV Inflammatory Cytokines Genes DNA Methylation

**Figure 4:**
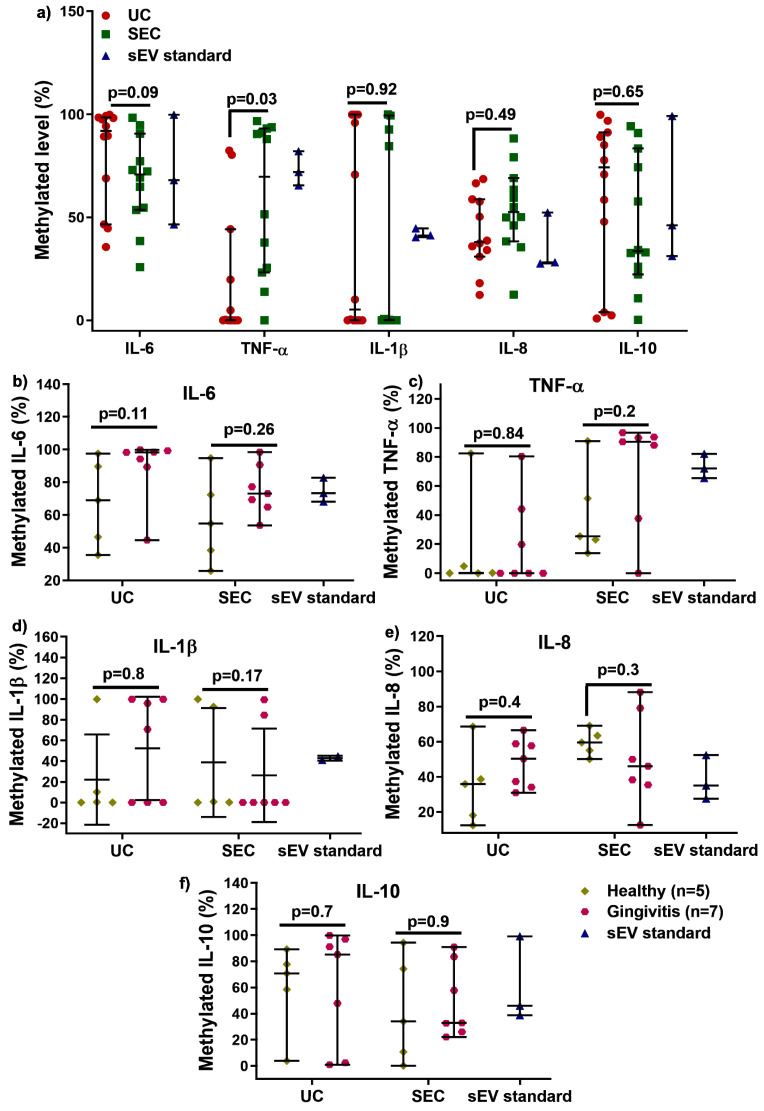
Comparison of UC and SEC on IL 6, TNF α, IL 1β, IL 8, and IL 10 gene promoter DNA methylation (**a**). The sEV DNA methylation in IL 6, TNF α, IL 1β, IL 8, and IL 10 for healthy (*n* = 5) and gingivitis (*n* = 7) for both UC and SEC (**b**–**f**).

We next examined the influence of UC and SEC DNA methylation changes for the inflammatory cytokines: IL−6, TNF-α, IL−1β, IL−8, and IL−10 (Figure 4). After bisulfite conversion, there were no significant differences in quantity and quality for converted sEV-associated DNA (Supplementary Figure S3). As shown in Figure 4a, there was no significant difference in IL−6, IL−1β, IL−8, and IL−10 methylation between UC and SEC, while SEC-DNA showed hypermethylation of TNF-α gene promoter compared to that of UC (only detected from five participants). There was no significant difference between healthy and gingivitis-sEV DNA methylation for all the tested genes for both UC and SEC.

### 2.5. Comparison of Pooled UC- and SEC-sEV on hGFs DNA Methylation 

**Figure 5:**
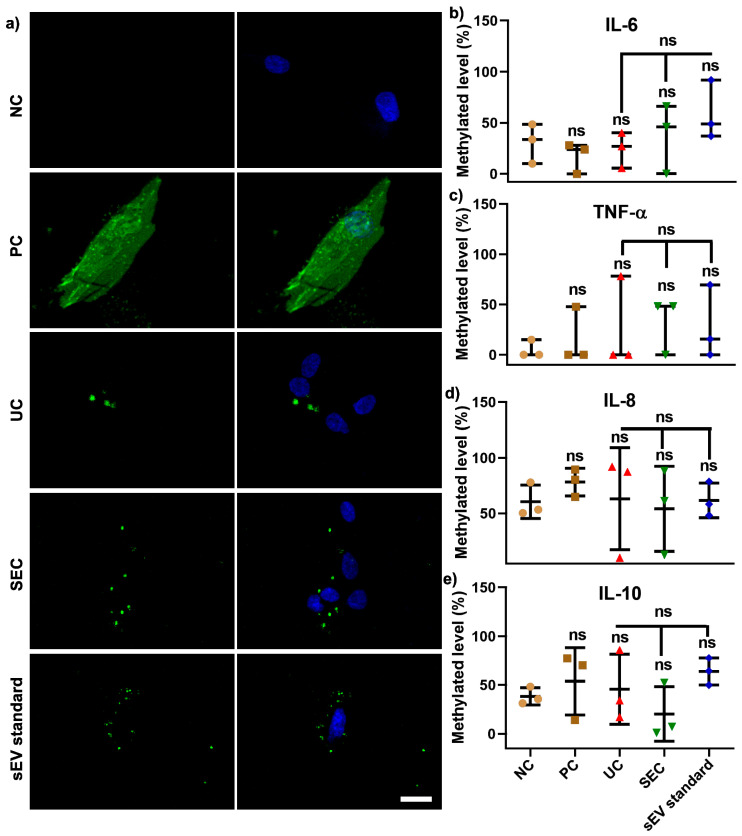
Pooled SEC-sEV and UC-sEV on hGFs uptake (**a**) and four inflammation cytokine gene promoters DNA methylation (**b**–**e**). Scale bar in (**a**): 20 μm. NC: negative control, PC: positive control-DiO labelled cells.

For function analysis, sEV isolated using both UC and SEC were pooled from 12 participants in order to investigate whether the different isolation methods can influence the effect on the recipient cell (hGFs) DNA methylation. The confocal images showed that DiO-labelled sEV from both the UC and SEC methods were primarily located around the nucleus when compared to a positive control (PC) with DiO-labelled cells (Figure 5a). Compared to hGFs without labelling (as negative control—NC), DiO labelling had no significant effect on cellular IL−6, TNF-α, IL−8, and IL−10 gene promoters methylation. The same trend was found in UC/SEC-sEV and sEV standard, and there was no significant difference between pooled UC-sEV and SEC-sEV uptake on the aforementioned four inflammatory cytokines genes DNA methylation (Figure 5b–e).

## 3. Discussion

This study demonstrated that the SEC method led to isolates that were more enriched in sEV particles than the UC method, without changing the methylation profile. This validates the SEC method as appropriate for sEV isolation for sEV subsequent methylation and epigenetic research.

### 3.1. Effect of UC and SEC Isolation Method on Salivary sEV Yield and Bacteria Detection

In this study, we evaluated the UC (“gold-standard” which takes ≈12 h) and SEC (a commercial SEC column which takes ≈1.5 h) methods for salivary sEV isolation in regard to sEV particles yield, morphology, protein content, and presence of oral-related bacteria. Interestingly, the SEC method had a significantly higher yield of sEV compared to UC. Furthermore, particle/protein ratio was significantly higher in SEC-sEV samples than in those from UC-sEV; while the morphology and mode values were comparable between the two methods. These findings are in line with other research reports showing that SEC led to higher yields of sEV compared to UC from both plasma [23] and urine [24].

Our pilot study demonstrated that sEV-associated genomic double-stranded DNA can be detected from salivary sEV using the Trizol method (Supplementary Figure S1); however, the purity of gDNA was not ideal (260/280 < 1.6). Therefore, optimizing sEV-DNA isolation is required to obtain DNA samples with improved purity.

The purity of sEV can be determined by the ratio of particle number to protein amount [25,26] and it has been suggested that highly pure vesicles show particle-to-protein ratios over 3 × 10^10^ particles per ug of protein [26]. In this study, both UC- and SEC-sEV led to impure sEV purity, however, SEC-sEV were purer than UC-sEV (2.6 × 10^8^ vs. 1.6 × 10^7^ particles/ug protein). These findings indicate that further isolation optimization for salivary sEV is required.

All species (i.e., bacteria) in the oral cavity and the host can secrete nano-scaled extracellular vesicles (EVs) that circulate in saliva [27]. Gram-negative bacteria secreted EVs are named outer membrane vesicles (OMVs), and they play a vital role in intracellular communication, microbial virulence, and host immune response [28]. However, most studies have overlooked the detection of Gram-negative bacteria from saliva sEV, such as those from the periodontal pathogens *T. forsythia, E. corrodens, P. gingivalis, P. anaerobius,* and *T. denticola*. Our study showed that UC and SEC had no significant effect on the isolation of gDNA from the five aforementioned periodontal pathogens present in saliva sEV. However, more in-depth research is required to investigate the isolation and characterization of bacterial OMVs. Overall, our data showed that the SEC method led to samples with enriched sEV particles, with no influence on periodontal pathogen detection, when compared to samples isolated using the UC method.

### 3.2. Utilizing a Commercial sEV Standard as a Control

This study used a commercial Salivary sEV standard (HansMed, Lonza) as a control (for the first time). We suggest that researchers should use a commercial sEV standard for all EV studies, for both quality control and to allow comparison between different studies, thus facilitating the translation of research findings towards clinical applications.

However, there is a limitation associated with using this salivary sEV standard in that self-reported health status of the participating donors does not necessarily indicate their true periodontal status. For instance, all 12 participants in our study were self-reported as ‘healthy’ without any systemic diseases; however, oral examination revealed that only five could be defined as periodontally ‘healthy’ (with BOP < 15%) and the remaining seven had gingivitis (with BOP > 15%) (Table 1), as defined in the most recent periodontal disease classification guidelines [21]. Further, it should also be considered that gingivitis is a common inflammatory condition associated with other systemic diseases, such as cardiovascular disease, adverse pregnancy outcomes, diabetes, rheumatoid arthritis, and osteoporosis (reviewed in [29,30]).

Thus, EV researchers should take periodontal status into account, particularly when they utilize participants with “self-reported” oral and/or systemic health status, since it is critical to compare research data with appropriate controls.

### 3.3. Healthy and Gingivitis-Derived sEV Characteristics—A Pilot Study

A recent study utilizing salivary sEV, isolated using an EQ kit, as potential biomarkers for periodontal disease status, suggested that CD9 and CD81 were decreased in periodontitis patient-derived sEV when compared with healthy controls [10]. This study did not include a gingivitis patient group, and a possible limitation is that EQ-sEV may contain soluble protein contamination. Our pilot study compared the UC and SEC methods for isolating salivary sEV from healthy (*n* = 5) and gingivitis (*n* = 7) patients, in relation to sEV particle size, concentration, size distribution, and periodontal bacterial pathogen detection. The results showed that the average mode of sEV and size distribution was comparable between healthy and gingivitis patients for both methods. However, UC-sEV particle concentration was significantly increased in gingivitis patients compared to healthy controls, while there was no change in sEV particles isolated by the SEC method between the healthy and gingivitis groups. Although it is unclear why only the UC isolation method leads to increased gingivitis-sEV particles compared to that from the healthy group, it may be reasonably assumed that gingivitis patients, who have gingival inflammation, have increased microbial concentrations. The NTA method is unable to distinguish between human and bacterial sEV. Indeed, this hypothesis can only be explored once pure microbial OMVs can be isolated from saliva. This pilot data suggests that UC-sEV particles concentrations could potentially be used as diagnostic markers for gingivitis, however, this needs to be investigated in a larger sample size.

Furthermore, there was no significant change in healthy and gingivitis-sEV regarding 16 s rRNA expression and periodontal pathogens (*T. forsythia*, *E. corrodens*, *P. gingivilas*, and *T. denticola)* for both methods.

The results demonstrated that in terms of comparing sEV from periodontally healthy and gingivitis patients, in terms of sEV particle size, size distribution, and periodontal bacterial pathogens, both UC and SEC methods performed in a similar manner.

### 3.4. UC and SEC on Five Inflammation Genes DNA Methylation in Salivary sEV

DNA methylation is one of the epigenetic modifications that are heritable and modulate downstream gene expression, without changing DNA sequences. In general, the addition of a methyl group to cysteine within the CpG regions of a specific gene leads to the suppression of gene expression [31]. The 5-mC methylation typically occurs at cytosine in CpG islands in vertebrates, where it is a region at least 200 bp long with greater than 50% GC content, and an observed-to-expected CpG ratio greater than 60% [32]. When present in promoters, 5-methylcytosine (5 mC) is associated with stable, long-term transcriptional silencing. This is crucial for normal development, abnormal expression of gene promoters leads to disorders, and hence this makes them a potential biomarker for disease progression and diagnosis [31,32]. Little is known about whether DNA methylation exists in salivary sEV. This study compared UC and SEC-sEV regarding the DNA methylation of five inflammatory cytokines genes (IL−6, TNFα, IL−1β, IL−8, and IL−10). The results demonstrated that the level of IL−6, IL−1β, IL−8, and IL−10 gene promoters DNA methylation was not affected by either the UC or SEC sEV isolation methods. However, the UC method identified lower levels (cannot be detected for seven participants) of TNF α promoter methylation in salivary sEV compared to both the SEC method and the control (Figure 4a), suggesting that the SEC method may be superior in terms of obtaining a representative sEV sample for assessing methylation.

The importance of epigenetic regulatory mechanisms in periodontal disease is emerging [15,33,34]. Our study demonstrated that there was no alteration in the five inflammatory cytokine genes DNA methylation in gingivitis compared to healthy patients, with the same outcome obtained with both UC- and SEC-sEV. This suggests that DNA methylation of inflammatory genes are tissue (or biofluid)-specific; thus, more candidate gene methylation profiles and whole genome-wide methylome profiles are required to determine the effectiveness of saliva for periodontal disease diagnostics.

To assess the toxicity and immunogenicity of sEV, our study compared the effect of pooled UC- and SEC-sEV from 12 participants on hGFs uptake and cellular inflammatory genes DNA methylation. As shown in Figure 5a, the DiO-labelled sEV were located around the nuclei for both UC and SEC-sEV and sEV standards, without alterations of IL−6, TNF-α, IL−1β, IL−8, and IL−10 gene promoter DNA methylation, compared with negative cells. This indicates that UC and SEC-sEV have minimal influence on cellular uptake and methylation in hGFs.

These findings are novel as DNA methylation of sEV samples obtained from different periodontal disease states has not been previously explored, and future research with a larger patient cohort is required to investigate whether salivary sEV associated DNA from gingivitis are hypomethylated (globally and specific genes) compared to that from healthy groups. Further, gingivitis, although highly prevalent, is a relatively mild and reversible condition, and greater differences in inflammatory gene methylation may be found in patients with the more destructive periodontal disease, periodontitis. Therefore, future investigations will include periodontitis patients of various severity.

## 4. Materials and Methods

### 4.1. Participant Recruitment and Saliva Sample Collection

Twelve participants aged 23–48 years (33.6 ± 2.09) were recruited from students and staff at the School of Dentistry, The University of Queensland, Australia. Comprehensive periodontal examinations were performed by two independent periodontists (to ensure data reliability) for each participant to determine their periodontal pocket depths (PPD) and bleeding of probing (BOP). This study was approved by the University of Queensland Human Ethics Research Committee (HREC No. 2018001225) and informed consent was obtained from all participants, who were non-smokers, had no underlying systemic diseases, and were not currently receiving any oral or periodontal treatment (details are shown in Table 1).

Unstimulated saliva samples were collected by asking participants to spit saliva directly into a 50 mL sterile centrifuge tube as described previously [17,35]. After refraining from eating and drinking for at least 1 h, the participants were asked to rinse with ≈10 mL of water to remove the food debris prior to saliva sample collection. Saliva samples were collected in the mornings between 9 am and 12 pm. Fresh saliva samples were aliquoted and frozen in a −80 °C freezer.

### 4.2. Salivary sEV Isolation

Salivary sEV were isolated using the UC method and SEC method from all 12 participants, according to the method described in Figure 1b. A commercial lyophilized salivary exosome (sEV) standard from ‘healthy’ donors (cat#: HBM-PESL; HansaBioMed, Lonza) was used as a control. HansaBioMed’s purified lyophilized exosomes are isolated through a combination of ultracentrifugation and microfiltration procedures.

#### 4.2.1. Salivary sEV Isolation Using the Differential Ultracentrifugation Method

The differential ultracentrifugation isolation method was used to isolate sEV from 1 mL of saliva as described previously [36], with details in Figure 1b. Briefly, 1 mL of saliva was diluted with 1 mL of Dulbecco’s phosphate-buffered saline (DPBS, Ca2+, Mg2+ free, ThermoFisher, Brisbane, Australia), and centrifuged at 800× *g* for 10 min, 2000× *g* for 30 min, and finally 12,000× *g* for 45 min at 4 °C to remove the cell debris and large EVs. The subsequent supernatant (2 mL) was transferred to an ultracentrifuge tube (Beckman, CA, Canada) and centrifuged at 100,000× *g* for 2 h (Type 70.1 Ti fixed ultracentrifuge rotor with adaptors [303376], Beckman, Canada). The 100,000× *g* pellet was suspended in 2 mL of PBS and centrifuged at 100,000× *g* for 2 h. Then the pellet containing the enriched sEV population was resuspended in 1 mL of PBS, prior to filtering through a 0.22 μm filter (Millipore, Brisbane, Australia). Vesicles were concentrated to ≈0.1 mL using an Amicon Ultra−4 Centrifugal Filter Unit (Merck Millipore, Brisbane, Australia) by centrifugation at 4000× *g* for 10 min at 4 °C.

#### 4.2.2. Salivary sEV Isolation Using SEC Method

Commercially available SEC columns (miniPURE, HansaBioMed, Lonza) were used to fractionate the saliva sample according to the manufacturer’s protocol (Figure 1b), as described previously [12]. Briefly, following 1:1 dilution with PBS, the samples were centrifuged at 300× *g* for 15 min, 1600× *g* for 15 min and 16,000× *g* for 20 min at 4 °C. The supernatant was loaded on an SEC column and 100 μL fractions were collected as indicated. Fractions 7 to 11 were collected and concentrated to ≈100 μL using an Amicon Ultra−0.5 Centrifugal Filter Unit (Merck Millipore, Brisbane, Australia) by centrifugation at 14,000× *g* for 5 min at 4 °C.

### 4.3. Salivary sEV Characterization

Following the recommendations from the International Society of Extracellular Vesicles [22], sEV were characterized according to morphology, sEV-associated protein content and size distribution, using transmission electron microscopy (TEM), Western blot, and Nanoparticle Tracking Analysis (NTA), respectively.

#### 4.3.1. Transmission Electron Microscopy (TEM)

For the TEM analysis, sEV samples were fixed in 3% (w/v) glutaraldehyde and analyzed as previously described [37]. Briefly, ≈5 μL of each sample was adsorbed on Formvar-carbon-coated and glow-discharged electron microscopy grids. After washing with PBS, the grids were transferred to a 50 μL drop of uranyl-oxalate solution, pH 7 for 3 min. The grids were imaged using an FEI Tecnai 12 transmission electron microscope (FEI, Hillsboro, OR, USA).

#### 4.3.2. Nanoparticle Tracking Analysis (NTA)

Nanoparticle tracking analysis was performed using a NanoSight NS500 instrument (NanoSight, Malvern, UK) using a 488 nm laser module and the NTA 3.1 software version, as previously reported [38]. Polystyrene latex beads (100 nm, Malvern, UK) were used as a sizing control and PBS as a negative control.

Samples were diluted 1:160 with Dulbecco’s PBS for the NS500 instrument to measure the rate of Brownian motion of nanoparticles in a light-scattering system. Five videos of 30 s were captured for each sample with a camera level of 14 and detection threshold at 5. Each video file was processed and analyzed to give the mean and mode of particle sizes, along with the concentration and the number of particles.

#### 4.3.3. Protein Content and Western Blot

Protein concentration in the sEV was determined using a Pierce BCA Protein Assay Kit (ThermoFisher Scientific) according to the manufacturer’s instructions. Absorbance was read at 595 nm on a Tecan Infinite spectrophotometer (Tecan, Männedorf, Switzerland).

Western blot, as previously described [39,40,41,42,43,44], was used to detect proteins enriched in sEV (CD 9, ALIX and TSG 101). To this end, the sEV protein samples (≈10 µg) were separated by SDS-PAGE and transferred to a polyvinylidene difluoride membrane using a Trans-Blot^®^ Turbo™ Transfer System (BioRad). The membrane was blocked with Odyssey^®^ Blocking Buffer (LI-COR^®^, Lincoln, NE) at room temperature for 1 h and primary antibodies (CD9, sc13118, 1:1000, Santa Cruz Biotechnology; ALIX, sc−53540, 1:1000, Santa Cruz Biotechnology and TSG101, 1:1000, Santa Cruz Biotechnology) were incubated overnight at 4 °C.

After washing with 0.1% *v/v* Tween 20 in TBS, anti-rabbit DyLight 800 secondary antibody (5151 S, Cell Signalling Technology, Danvers, MA) and anti-mouse DyLight 700 (5257 S, Cell Signalling Technology) secondary antibody was diluted 1:15,000 in Odyssey Blocking Buffer (LI-COR^®^) and incubated for 1 h. The blots were visualized using an LI-COR Biosciences Odyssey IR Imaging System and data quantified using Image Studio software version 4.0.

### 4.4. sEV DNA and RNA Isolation by Trizol Method

Total RNA and genomic double-stranded DNA (gDNA) was isolated from ≈10^9^ sEV particles using the Trizol method, following the manufacturer’s instructions. Total RNA and gDNA were extracted as described previously [17]. The quality and quantity of RNA and gDNA were measured using a Tecan Infinite M200 Pro Spectrophotometer (TECAN, Männedorf, Australia).

Quantitative real-time PCR was performed to determine the bacterial gDNA in salivary sEV samples as described previously [17]. The reaction was comprised of 5 μL of 2 × PowerUp SYBR Green Master Mix (ThermoFisher Scientific, Brisbnae, Australia), 100 μM of forward and reverse primers, and 200 pg of gDNA template. qPCR reaction (10 μL) was performed in StepOnePlus PCR equipment (Applied Biosystems, Brisbane, Australia) according to the manufacturer’s instructions. Additionally, sEV gDNA-qPCR was carried out for the periodontal pathogens (*T. forsythia, E. corrodens, P. gingivalis, P. anaerobius,* and *T. denticola*) detection.

### 4.5. DNA Bisulfite Conversion and Quantitative Methylation-Specific Polymerase Chain Reaction (qMSP)

Isolated sEV gDNA (500 ng) was bisulfite-converted with an EpiJET Bisulfite Conversion Kit (ThermoFisher Scientific, Australia). Modified DNA was resuspended in 15 μL of distilled water and stored at −20 °C. Quality and quantity of converted DNA (resembles RNA) were measured for RNA with a Tecan Infinite M200 Pro Spectrophotometer.

The qMSP primer pairs of periodontium-related inflammatory genes (IL−6, TNF-α, IL−1β, IL−8, and IL−10) were designed around the CpG islands and the transcription start site (TSS) by the online tool MethPrimer (http://www.urogene.org/methprimer/) [17,45]. Both methylation and unmethylation primers for each gene were assessed using gDNA and were found not to amplify, confirming the specificity of the primer pairs used in this study.

qMSP was performed in StepOnePlus PCR equipment (Applied Biosystems, Brisbane, Australia), consisting of 2 × PowerUp SYBR Green Master mix (ThermoFisher Scientific, Australia), 100 μM of forward and reverse primers, and 200 pg of converted DNA template. The methylated CTm value was normalized against the unmethylated CTu value, where ΔCT = CTm − CTu. The relative methylation expression was calculated as 2−ΔCT [18]. The relative methylated level (%) of total CpG islands was calculated using the following formula: $\frac{2-\Delta \mathrm{CT}}{2-\Delta \mathrm{CT}+1}$ × 100%.

### 4.6. Salivary sEV Labelling and Uptake by Human Primary Gingival Fibroblasts

#### 4.6.1. Human Primary Gingival Fibroblasts Cell Culture

Human primary gingival fibroblasts (hGFs) were isolated and cultured as previously described [19], following ethics approval by the University of Queensland Human Research Ethics Committee (approval number: 2019000134), Brisbane, Australia. Briefly, gingival marginal tissue was collected from three healthy female subjects (age: 18, 24, and 37 years) who underwent third molar extractions.

For the establishment of hGFs culture, the extracted marginal gingival tissue was dissected into small pieces and placed into a tissue culture flask to allow the explant culture. Then, the cells were cultured in low glucose Dulbecco’s Modification of Eagle’s Medium (DMEM) (Gibco) supplemented with 10% Fetal Bovine Serum (FBS, ThermoFisher Scientific) and Antibiotic-Antimycotic (ThermoFisher Scientific) containing 100 units/mL of penicillin, 100 µg/mL of streptomycin, and 0.25 µg/mL of Amphotericin B. Subsequent subcultures were maintained in DMEM containing 10% FBS, 50 units/mL penicillin, and 50 μg/mL streptomycin at 37 °C in a 5% CO2 incubator. Cells from three donors (*n* = 3) at passage 5 were used in this study.

#### 4.6.2. sEV Labelling, hGFs Uptake, and Cellular qMSP

To compare UC and SEC derived sEV uptake in hGFs, ≈4 × 10^7^ sEV particles from each participant (*n* = 12) were pooled and labelled with 3, 3′-Dioctadecyloxacarbocyanine perchlorate (DiO, Sigma) at a concentration of 5 μg/mL. After labelling with DiO (green) at room temperature for 1 h, unstained DiO was removed with an Amicon Ultra−0.5 Centrifugal Filter Unit (10 kDa, Merck Millipore) by centrifugation at 14,000× *g* for 5 min at 4 °C, as previously described [46,47].

For confocal imaging, DiO-labelled sEV (3.8 × 10^7^ particles) were incubated with hGFs (1500 cells in suspension) for 5 min prior to placement into a well of a 96-well plate. After 4 h incubation, cells were washed with PBS to remove unbound labelled sEV and fixed with 4% PFA for 5 min. Cells stained with DiO was used as a positive control (PC). Cells without labelling were used as a negative control (NC). The nuclei were stained using DAPI and images were acquired with a Nikon Confocal Microscope (Nikon, Tokyo, Japan).

For cellular qMSP analysis, gDNA was isolated from hGFs after 4 h incubation with DiO-labelled sEV and qMSP was performed for IL−6, TNF-α, IL−1β, IL−8, and IL−10 gene promoters as described previously [17].

### 4.7. Statistical Analysis

All data are presented as median ± 95% CI. A paired non-parametric *t*-test analysis (Wilcoxon) was performed to compare data of UC- and SEC-sEV from the same participant. Non-paired non-parametric *t*-test (Mann–Whitney) was carried out for data from healthy and gingivitis patients. A value of *p* < 0.05 was considered statically significant.

### 4.8. EV-TRACK

We submitted all relevant data of our experiments to the EV-TRACK knowledgebase (EV-TRACK ID: EV200019) [48].

## 5. Conclusions

Time-efficiency, protein contamination, initial biofluid volume, and effect on functional assays are critical parameters to consider when choosing an sEV isolation method. Taken together, our findings indicate that the SEC method is appropriate for salivary sEV isolation, especially in terms of time-efficacy, higher yield, and enhanced quality. Both UC and SEC methods had a minimal effect on five inflammatory cytokine DNA methylation genes for salivary sEV-associated DNAs and biological function on cellular inflammatory cytokine DNA methylation in hGFs. This study is an important initial step in the field of salivary sEV DNA epigenetics research for the diagnosis of periodontal disease status, and further research, such as sEV methylome profile, is required to advance the knowledge in sEV compositions and functions. It is also recommended that EV researchers utilize an sEV standard for biomarker discovery research, in order to standardize research outputs and facilitate translational outcomes. Additionally, we propose that periodontal health is taken into account when utilizing self-reported ‘healthy’ controls.

## Figures and Tables

**Table 1:** Participant data.

Gender	Age	PPD	BOP	Race
F	33	<4 mm	27%	Asian
M	28	<4 mm	41%	Asian
M	39	<4 mm	14%	Other
F	24	<4 mm	39%	Caucasian
M	40	<4 mm	39%	Asian
F	26	<4 mm	26%	Caucasian
F	38	<4 mm	10%	Asian
M	24	<4 mm	6%	Caucasian
M	34	<4 mm	49%	Caucasian
M	36	<4 mm	14%	Asian
M	34	<4 mm	70%	Caucasian
M	48	<4 mm	14%	Caucasian

Abbreations: PPD: periodontal probing depth; BOP: bleeding on probing.

**Table 2:** Detailed Nanoparticle Tracking Analysis (NTA) information (mean ± SD).

	UC-sEV	SEC-sEV
sEV particles/mL saliva	5.07 × 10^9^ ± 2.9 × 10^9^	2.7 × 10^10^ ± 2.2 × 10^10^
sEV particles/mL saliva: <50 nm	2.8 × 10^5^ ± 5.7 × 10^5^	1.1 × 10^5^ ± 2.8 × 10^5^
sEV particles/mL saliva: 50–150 nm	1.1 × 10^7^ ± 9.9 × 10^6^	6.6 × 10^8^ ± 6.9 × 10^8^
sEV particles/mL saliva: 150–200 nm	1.2 × 10^7^± 8.3 × 10^6^	1.3 × 10^9^ ± 9.9 × 10^8^
sEV particles/mL saliva: >200 nm	1.9 × 10^6^ ± 1.1 × 10^6^	1.9 × 10^8^ ± 1.6 × 10^8^
sEV particles per μg protein	1.6 × 10^7^ ± 9.4 × 10^7^	2.6 × 10^8^ ± 1.5 × 10^8^

## Supplementary Materials

Supplementary Materials can be found at https://www.mdpi.com/1422-0067/21/15/5273/s1.
