# Supplementary material for: Detection of Salivary Small Extracellular Vesicles Associated Inflammatory Cytokines Gene Methylation in Gingivitis
Source: Int J Mol Sci. 2020 Jul 24;21(15):5273. doi: 10.3390/ijms21155273 (PMC7432462; doi:10.3390/ijms21155273)
Supplement: Supplementary file 1 [file ijms-21-05273-s001.docx]

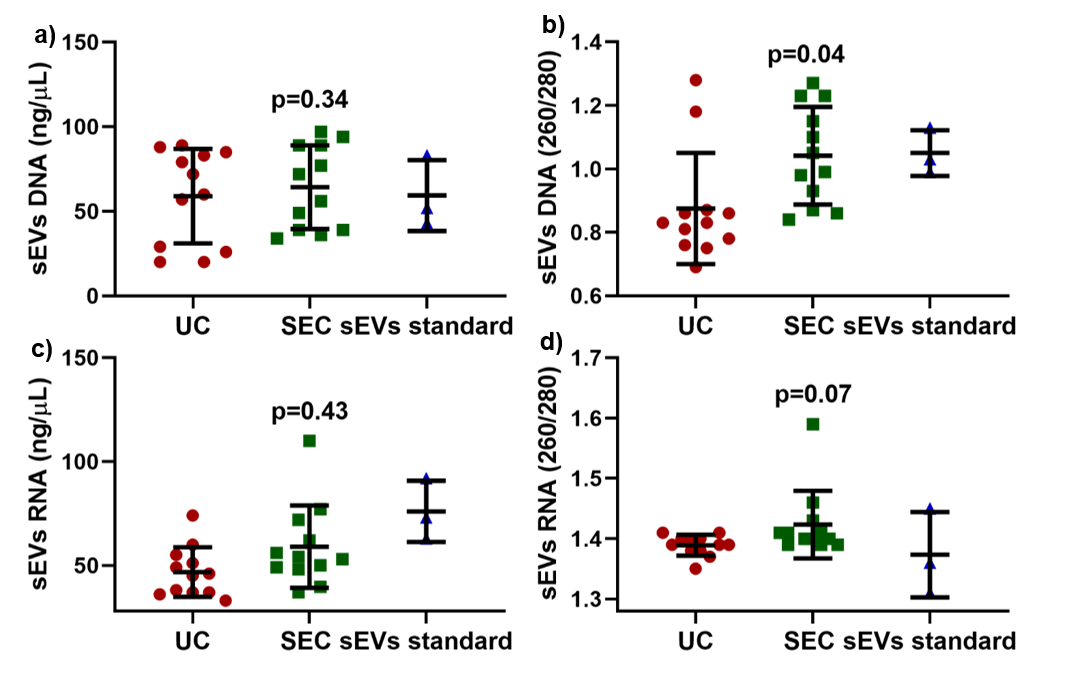


**Supplementary Figure S1:** Salivary sEVs-associated dsDNA concentration (**a**), quality (**b**) and RNA concentration (**c**) and quality (**d**).


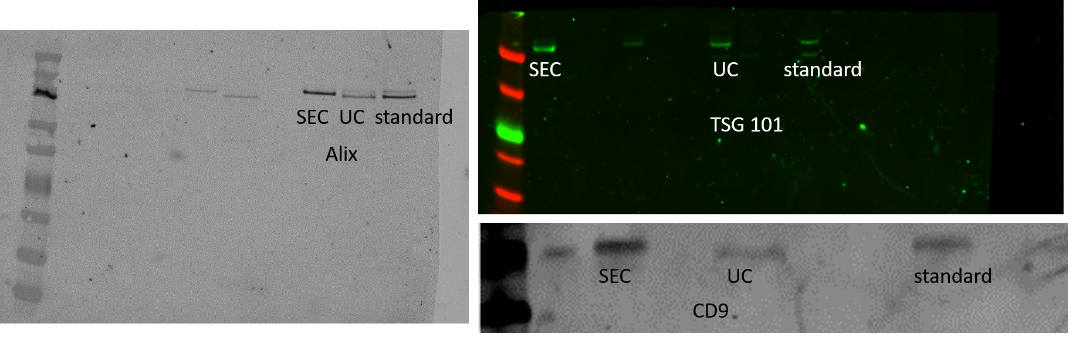


**Supplementary Figure S2.** The raw images of Western Blot for Figure 2 g.


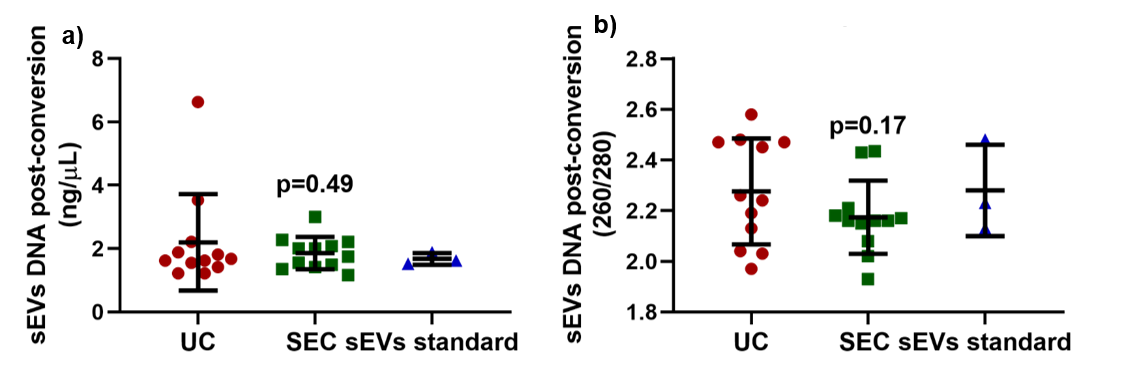


**Supplementary Figure S3.** Salivary sEVs DNA post-bisulfite conversion concentration (**a**) and quality (**b**) from 500 ng of DNA template. Post-bisulfite conversation, the samples were measured as RNA due to their resemblance of single-stranded RNA; thus, the 260/280 ratio is >2.0.
